# The Effect of Mitomycin-C on Corneal Endothelial Cells after Photorefractive Keratectomy

**Published:** 2011-01

**Authors:** Mohammad Zare, Mohammad-Reza Jafarinasab, Sepehr Feizi, Mitra Zamani

**Affiliations:** Ophthalmic Research Center, Labbafinejad Medical Center, Shahid Beheshti University of Medical Sciences, Tehran, Iran

**Keywords:** Myopia, Photorefractive Keratectomy, Mitomycin-C, Endothelial Cell Density, Mean Cell Area, Coefficient of Variation in Cell Size

## Abstract

**Purpose:**

To evaluate short-term changes in central corneal endothelial cell density and morphology after photorefractive keratectomy (PRK) with mitomycin-C (MMC) 0.02% in patients with moderate myopia.

**Methods:**

In this prospective interventional case series, patients with moderate myopia (spherical equivalent refractive error from −4.0 to −8.0 D) underwent PRK with a single intraoperative application of MMC 0.02% for 40 seconds. Specular microscopy was performed preoperatively and repeated 6 months after surgery to determine changes in central corneal endothelial cell density (ECD), mean cell area (MCA) and coefficient of variation in cell size (CV).

**Results:**

Overall, 42 eyes of 21 participants with mean age of 26.2±6.3 years underwent surgery. Mean preoperative spherical equivalent refractive error was −5.2±1.2 D which was reduced to −0.4±0.5 D postoperatively (P < 0.001). Mean ECD was reduced insignificantly from 2,920±363 cells/mm^2^ preoperatively to 2,802±339 cells/mm^2^ postoperatively (P = 0.59). Similarly, there was no significant change in MCA or CV at six months (P = 0.76 and 0.52, respectively).

**Conclusion:**

Application of MMC 0.02% for 40 seconds during PRK in patients with moderate myopia did not significantly affect central corneal endothelial cell density and morphology after a 6 month follow up period.

## INTRODUCTION

Mitomycin-C (MMC) was first isolated from cultures of Streptomyces caespitosus by Hata in 1956.^1^ Since then, it has been widely used intraoperatively for pterygium excision, trabeculectomy, and surface ablation keratorefractive procedures. Additionally, it is used topically in a cyclic fashion for primary or recurrent ocular surface squamous neoplasia.^2^ Having been found safe and effective in animals, MMC was suggested for application during surface ablation procedures to reduce postoperative haze formation.^3^,^4^ It can effectively reduce haze formation and hence, improve the predictability of visual outcomes following refractive surgery. Despite these advantages, MMC can potentially damage all three main corneal cell types including epithelial (differentiated epithelium and limbal cells), stromal (keratocytes), and endothelial cells.

Several studies have investigated the effect of a single intraoperative dose of MMC during refractive surgery on the corneal endothelium. Some clinical^5^,^6^ and laboratory^7^–^9^ studies have reported significant corneal endothelial toxicity. However, the majority of clinical studies have reported no significant change in corneal endothelial density or morphology with follow-up period ranging from 3 to 18 months.^10^–^15^ Most studies on MMC have employed a short duration of exposure, less than 20 seconds. Since different durations of MMC application have been used, discrepancies in the findings of these studies make it difficult to reach a definite conclusion regarding the safety of MMC for corneal endothelial cells.

Herein, we evaluate changes in central corneal endothelial cell density and morphology in a group of participants with moderate myopia after photorefractive keratectomy with MMC application for 40 seconds.

## METHODS

This prospective interventional case series included 42 eyes of 21 patients (16 female subjects) aged 18 to 46 years with moderate myopia (spherical equivalent, −4.0 to −8.0 D). Contraindications for refractive surgery, such as severe dry eye, untreated blepharitis, corneal scarring, keratoconus, corneal dystrophy or degeneration, lens opacity, glaucoma, or retinal diseases, were considered as exclusion criteria. Additionally, history of ocular trauma or surgery led to patient exclusion. The Ethics Committee approved the study and informed consent was obtained from all participants after explaining the purpose of the study.

Preoperative evaluations included a case history, measurement of uncorrected visual acuity (UCVA) and best spectacle-corrected visual acuity (BSCVA) using the Snellen chart, manifest and cycloplegic refraction, slitlamp examination of the anterior segment, dilated fundus examination, measurement of intraocular pressure, central corneal pachymetry using an ultrasonic contact probe (A/B scan; Sonomed Inc., Lake Success, NY, USA), and corneal topography (Orbscan II, Orbtech, Bausch & Lomb, Rochester, NY, USA). Specular microscopy was also performed (SP 2000P, Topcon Inc., Tokyo, Japan) to determine central corneal endothelial cell density (ECD), mean cell area (MCA), and coefficient of variation in cell size (CV). To reduce sampling error, the clearest specular image containing at least 100 endothelial cells in the center of the field was used.^16^

### Surgical Technique

All participants were operated on by a single ophthalmologist (MRJ) in a private practice setting, using a flying spot excimer laser machine (Technolas 217z, Bausch & Lomb Surgical, Rochester, NY, USA), with an emission wavelength of 193 nm, fixed pulse repetition rate of 50 Hz, and radiant exposure of 400 mJ. Topical tetracaine hydrochloride 0.5% eye drops were used to anesthetize the cornea. Antisepsis was performed by applying 10% povidone-iodine solution to the skin of the eyelids and periocular area for 1 minute and the eyes were washed out by 20 mL of balanced salt solution (BSS). The epithelium was mechanically removed in the 8.0 mm central cornea. Thereafter, ablation was performed using the Planoscan software (Bausch & Lomb, Rochester, NY, USA). Optical zone was 6.0 mm and manifest refractive error was considered as the target for correction in all cases. Subsequently, a sponge, 7.0 mm in diameter, soaked with mitomycin-C 0.02% (0.2 mg/mL, diluted in BSS), was applied over the ablated surface for 40 seconds. This was followed by copious irrigation with BSS, and fitting a contact lens (OmniFlex; Hydron, Fareham, UK) at the conclusion of the operation.

### Follow-up

Postoperatively, all participants were given chloramphenicol 0.5% eye drops every 6 hours for 1 week. Betamethasone 0.1% eye drops were initially administered twice a day until complete corneal reepithelialization. Thereafter, the dose was increased to four times a day for the next 2 weeks and then gradually tapered over 6 weeks. Once corneas completely reepithelialized (usually between 3 to 5 days), the contact lens was removed.

Follow-up examinations were scheduled 1, 3, 7, and 30 days and 1, 3, and 6 months postoperatively. At each examination except on days 1 and 3, UCVA, BSCVA, manifest refraction, and tonometry were checked. Specular microscopy was repeated 6 months after surgery.

### Statistical Analysis

Data were presented as mean ± standard deviation. Paired t-test was used to compare pre- and postoperative refractive error, ECD, MCA, and CV. Multiple linear regression analysis was performed to investigate whether the amount of correction and ablation depth had any correlation with reduction in postoperative ECD. P-values less than 0.05 were considered as statistically significant.

## RESULTS

Forty-two eyes of 21 patients with mean age of 26.2±6.3 (range, 18 to 46) years underwent surgery. Mean preoperative spherical equivalent refractive error was −5.2±1.2 (range, −4.0 to −8.0) D which was reduced to −0.4±0.5 (range, −1.25 to +0.5) D postoperatively (P<0.001). There was no significant difference between pre- and postoperative BSCVA (0.05±0.13 logMAR and 0.04±0.14 logMAR, respectively; P=0.57). Mean preoperative central corneal thickness and ablation depth were 569.4±38.5 (range, 497 to 653) μm and 98.9±19.7 (range, 63.4 to 122) μm respectively. Six months after the operation, mean ECD was reduced by 4.0% (P=0.59) from 2,920±363 (range, 2,079 to 3,893) cells/mm^2^ preoperatively, to 2,802±339 (range, 2,113 to 3,434) cells/mm^2^. The average difference between pre- and postoperative ECD was 29.47±296.2 (range, −537.0 to 775.0) cells/mm^2^. Ten out of 42 eyes (23.8%) experienced reduction exceeding 100 cells/mm^2^, while 11 eyes (26.2%) had an increase of 100 cells/mm^2^ or more. There was no significant difference between these two subgroups in terms of preoperative refractive error, central corneal thickness, or ablation depth. There was no significant change in MCA [352±54 (range, 256 to 530 μm^2^) preoperatively versus 362±45 (range, 291 to 473 μm^2^) postoperatively, P=0.76] or CV [22.2±6.8 (range, 11 to 38) vs. 23.5±4.6 (range, 15 to 31), P=0.52]. Multiple regression analysis revealed no significant correlation between reduction in ECD and ablation depth (P=0.75) or spherical equivalent refractive error (P=0.20).

None of the patients developed postoperative complications such as persistent epithelial defect, infectious keratitis, or significant haze formation. Additionally, enhancement procedures for residual refractive errors were not required in any of the participants.

## DISCUSSION

In the current study, reduction in cell density was 4.0%, which is exactly similar to a 4.1% variation previously observed in repeated measurements of endothelial cells in non-operated eyes.^17^ Furthermore, 26.2% of the patients experienced an increase of 100 cells/mm^2^ or more in endothelial cell counts which may be attributed to variations observed in repeated measurements. There was no significant change in the morphological features of the endothelial cells including mean cell area and coefficient of variation in cell size. The results of this study are supported by many others reporting no measurable effect on endothelial cell density or morphology after a single intraoperative application of MMC 0.02% ranging from 12 seconds to 2 minutes during surface ablation. Evaluating retrospectively a large number of eyes undergoing PRK with MMC 0.02%, Lee et al^10^ found that endothelial cell density remained unchanged after 16 months. In a prospective interventional study, Zhao et al^15^ found no significant change in central endothelial cell density or morphology at least 6 months after a 15 second application of MMC 0.02%. Additionally, they did not observe any significant correlation between ablation depth and changes in endothelial cell density or morphology. Another study comparing ECD after LASEK with or without MMC 0.02% application for 30 seconds, showed no significant difference between the two groups.^12^ Similarly, other prospective studies applying MMC 0.02% after surface ablation reported no significant change in cell density or morphology with follow-up ranging from 3 to 18 months.^11^–^15^

In contrast, there are two studies associating MMC use with significant corneal endothelial cell loss. Morales et al^5^ conducted a randomized clinical trial on 18 eyes of 9 participants with myopia between −1.75 and −6.25 D. Fellow eyes were randomly assigned to PRK with MMC 0.02% or BSS for 30 seconds, the authors reported a significant reduction in endothelial cell count at month 1 (14.7%) and month 3 (18.2%) in the MMC group, while corresponding figures were not significantly changed in the BSS group (4.3% and 5.0%, respectively). The sample size in the mentioned study was too small to draw a conclusive result. In another prospective non-randomized study, Nassiri et al^6^ used MMC 0.02% for 10 to 50 seconds in eyes undergoing PRK with an ablation depth of 75 μm or more and observed a significant decrease in central endothelial cell density. This decrease, which was documented during the first postoperative week, continued at a slower pace up to 6 months. Additionally, the authors reported the duration of MMC application and male sex to be significantly associated with greater endothelial cell loss.

The clinical significance of the results of these two studies^5^,^6^ and laboratory studies^7^–^9^ documenting MMC toxicity for endothelial cells is not clear as there is no report of corneal decompensation after surface ablation and MMC application since it was introduced for refractive surgery in 1991. Although there are case reports describing corneal decompensation with MMC,^18^–^21^ mainly after glaucoma surgery or phototherapeutic keratectomy, at least one reason other than MMC application was present for the compromised endothelium in each report, including previous cataract and glaucoma surgery,^18^ chronic uveitis^19^, high intraocular pressure,^19^,^20^ or complicated surgery^21^. Therefore, it remains unclear whether the corneal decompensation was caused by the use of mitomycin-C per se.

The decrease in keratocyte density after MMC application is correlated with its concentration and to a lesser extent exposure time.^22^ A similar association can be expected for the effect of MMC on endothelial cells. Theoretically, another determinant could be the depth of ablation; deeper ablation leaves a thinner residual stroma, allowing the drug to penetrate and concentrate in the anterior chamber to a higher concentration. However, MMC 0.02% has been applied for 12 seconds to 2 minutes after surface ablation, depending on ablation depth, and has been found to be safe for endothelial cells. The results of the current study show that the amount of correction and hence, ablation depth had no significant correlation with ECD. This observation is supported by the results of Zhao et al^15^. Therefore, the conventional residual stromal thickness set to prevent iatrogenic keratectasia (> 300 μm) seems to be enough to protect the endothelium against the adverse effects of topical MMC.

In summary, this study demonstrated that intraoperative application of MMC 0.02% for 40 seconds in a group of participants with moderate myopia did not adversely affect endothelial cell density and morphology up to 6 months.
